# Calibrating ADL-IADL scales to improve measurement accuracy and to extend the disability construct into the preclinical range: a systematic review

**DOI:** 10.1186/1471-2318-11-42

**Published:** 2011-08-16

**Authors:** Robert A Fieo, Elizabeth J Austin, John M Starr, Ian J Deary

**Affiliations:** 1 Centre for Cognitive Ageing and Cognitive Epidemiology, Department of Psychology, University of Edinburgh, UK; 2 Department of Psychology, University of Edinburgh, UK; 3 Centre for Cognitive Ageing and Cognitive Epidemiology, Geriatric Medicine Unit, University of Edinburgh, Royal Victoria, UK

## Abstract

**Background:**

Interest in measuring functional status among nondisabled older adults has increased in recent years. This is, in part, due to the notion that adults identified as 'high risk' for functional decline portray a state that is potentially easier to reverse than overt disability. Assessing relatively healthy older adults with traditional self-report measures (activities of daily living) has proven difficult because these instruments were initially developed for institutionalised older adults. Perhaps less evident, are problems associated with change scores and the potential for 'construct under-representation', which reflects the exclusion of important features of the construct (e.g., disability). Furthermore, establishing a formal hierarchy of functional status tells more than the typical simple summation of functional loss, and may have predictive value to the clinician monitoring older adults: if the sequence task difficulty is accelerated or out of order it may indicate the need for interventions.

**Methods:**

This review identified studies that employed item response theory (IRT) to examine or revise functional status scales. IRT can be used to transform the ordinal nature of functional status scales to interval level data, which serves to increase diagnostic precision and sensitivity to clinical change. Furthermore, IRT can be used to rank items unequivocally along a hierarchy based on difficulty. It should be noted that this review is not concerned with contrasting IRT with more traditional classical test theory methodology.

**Results:**

A systematic search of four databases (PubMed, Embase, CINAHL, and PsychInfo) resulted in the review of 2,192 manuscripts. Of these manuscripts, twelve met our inclusion/exclusion requirements and thus were targeted for further inspection.

**Conclusions:**

Manuscripts presented in this review appear to summarise gerontology's best efforts to improve construct validity and content validity (i.e., ceiling effects) for scales measuring the early stages of activity restriction in community-dwelling older adults. Several scales in this review were exceptional at reducing ceiling effects, reducing gaps in coverage along the construct, as well as establishing a formal hierarchy of functional decline. These instrument modifications make it plausible to detect minor changes in difficulty for IADL items positioned at the edge of the disability continuum, which can be used to signal the onset of progressive type disability in older adults.

## Background

In the U.S., the number of those aged 65+ in the year 2000 was approximately 35 million. In 2050, this figure is expected to rise to nearly 82 million [1]. The potential burden to healthcare becomes apparent if we couple these figures with evidence indicating that 55 years-of-age is the median age of detectable chronic disability [2]. Such forecasts have prompted gerontologists and geriatricians to consider more seriously prevention-type models, with an emphasis on the earliest stages of functional decline. Increased interest in the maintenance of function and prevention of disability has led to relatively new diagnostic criteria, such as symptoms of frailty or preclinical disability. The utility of identifying individuals who are 'high risk' for future functional decline rests on the notion that it is potentially an easier state to reverse than overt disability [3]. Intervention programs designed to prevent functional decline in older adults show that participants with relatively good functional status or moderate frailty are those who benefit the most from these programs [4]. However, 'prehabilitation' strategies necessitate the use of assessment measures that exhibit a high degree of sensitivity. Standardised tests of physical performance have been employed with increasing frequency in recent years, presumably to meet this demand for greater sensitivity [5].

Activities of Daily Living (ADL) [6] and Instrumental Activities of Daily Living (IADL) [7] were developed to assess capabilities relating to the maintenance of self and lifestyle, which often includes self-care, keeping one's life-space in order, and obtaining resources [8]. When compared to performance-based measures (e.g., walk time), ADLs and IADLs generally display weak face validity, reproducibility, and sensitivity to change [9]. Also, as the emphasis has changed toward early detection in community-dwelling older adults, for whom dependency in self-reported ADL-IADLs is uncommon, researchers often have to cope with large ceiling effects, in which greater than 90% of subjects endorse no 'difficulty' or 'dependency' on ADL tasks [10]. It has been proposed that the relative standing of ADL-IADLs could be enhanced by improving construct validities to levels that are at least equivalent to those of physical performance measures [11]. Enhancements of this nature have progressed relatively slowly. The justification for improving construct validity in ADL-IADLs, rather than abandoning them in favour of performance measures, can be found in two observations. First, there is evidence that self-reported ADL-IADLs and performance based measures are comparable to each other, but usually measure different aspects of functioning [5]. Second, combining information from self-report and performance measures has been shown to increase prognostic value, particularly in high-functioning older adults [10].

One reason given as to why the psychometric properties of self-reported ADL-IADLs can be insufficient pertains to the ordinal nature of Likert scoring methods. This traditional, and still the most common, aggregate method of scoring computes a raw total score by summing responses to individual items. Despite the popularity of the aggregate scoring method, there are well-established problems with raw scale scores that make them difficult to interpret [12]. One problem pertains to weighing each item equally; the total score method assumes that each item or symptom on the scale represents an equal level of severity, which is almost never true [13]. Furthermore, the two methods (i.e., IRT vs. Likert scoring), with respect to difficulty ranks, can diverge considerably. For example, it has been demonstrated, within a 16-item scale, five Likert items scores differed by three or more ranks compared to Partial Credit (Rasch model) scores [14].

Revised ADL-IADLs, through the use of Item Response Theory (IRT), avoid the pitfalls of aggregated approaches to self-reported disability. In contrast to traditional summative scoring methods, IRT models meet the conceptual requirements of order and additivity [15]. This is primarily achieved by converting the ordinal level data into interval level log-odd units, which are computed for both items and person separately and then placed on a common scale [16]. "With the priority placed on establishing interval units of measure, the investigator derives complementary tools for understanding the nature of scale's meaning and, more importantly, provides a substantive context within which an individual's score on a scale may be interpreted" [[17], p.52]. Establishing interval level units permits one to identify important features of the construct that have been excluded. These gaps in measurement (typically referred to as construct under-representation) are worth investigating because they are thought to undermine construct validity. This means that there may be uneven rates of change in the construct being measured. For instance, an increase in a 10-point scale can represent different amounts of improvement at different parts of the functional status scale; it might be more difficult for a person to improve from 9 to 10 than from 4 to 5 [18].

Construct validity for ADL-IADL scales can also be enhanced by formally confirming a hierarchy of decline. For example, by supporting or refuting the expectation that 'Stepping over obstacles' is a more challenging task than 'Walking over a level surface' [19]. Establishing a hierarchy of functional decline tells more than the typical simple summation of functional loss, and may have predictive value to the clinician monitoring older adults: if the sequence is accelerated or out of order it may indicate the need for interventions [20]. IRT-based transformations allow for items to be ranked unequivocally on a hierarchy based on item difficulty, ranking items from easiest to most difficult [21]. Ordering items or tasks by group mean scores does not imply that this ordering also holds at the individual level. "Any set of items can be ordered by item mean scores, but whether such ordering also holds for individuals has to be ascertained by means of empirical research. Only when the set of items has an invariant item ordering (IIO) can their cumulative structure be assumed to be valid at the lower aggregation level for individuals" [[22], p.579].

In addition to improving the validity of ADL-IADL measures by reducing ceiling effects, identifying construct under-representation, and confirming a formal item hierarchy, IRT methods can expand upon classical approaches to instrument reliability. Knowing the instrument's reliability provides information about the variance or error associated with the person's true score. The true score refers to the average score a person would receive if they were tested repeatedly (necessarily hypothetical) [23]. Instrument reliability relating to disability can tell us whether observed changes are due to, for example, an intervention aimed at attenuating severity or problems with the precision of an instrument. An unreliable disability instrument may therefore underestimate the size of the benefit obtained from an intervention. IRT enhances interpretive power by providing measurement precision that varies with a person's ability level [24]. This information (i.e., error that varies by person performance) can be used to identify the most sensitive part of the instrument or scale under investigation [25]. Whereas in CTT a single number (e.g., the internal-consistency reliability coefficient, or the SEM based on that reliability) would be used to quantify the measurement-precision of a test, a continuous function is required in IRT to convey comparable data [26].

The goal of this systematic review is to identify manuscripts that use Item Response Theory to revise or develop ADL-IADL scales used for community-dwelling older adults. These revised scales should: (i) assess internal validity (cause and effect) by formally confirming a hierarchy of functional decline; (ii) enhance content validity, i.e., reduce ceiling effects to thresholds approaching 15%; and (iii) quantify construct under-representation (i.e., gaps in coverage) by converting the raw aggregated disability score into interval level measurement. The by-product of the aforementioned goals will be the identification of ADL-IADL instruments that are highly sensitive to the early stages of disability, and more accurate in detecting change over time. Lastly, this review is not concerned with establishing the superiority of one method over another (i.e., item response theory vs. classical test theory) in relation to scale analysis.

## Methods

### Data sources

Published studies were identified through searches of PubMed (from its inception in January 1966 until November 2008), PsychInfo (1872 until November 2008), Embase (1980 until November 2008) and CINAHL (1981 until November 2008) databases. Keyword, title and abstract information were used. The main search terms included 'functional decline' or 'function* (the symbol is used for identifying all words starting with function, e.g., functional, functions) status' or IADL or 'instrumental activities of daily living' or ADL or 'activities of daily living' or BADL or 'basic activities of daily living' or 'personal activities of daily living' or 'functional disability' or 'functional tasks' or 'loss of independence' or disabled or disabilit* or 'functional impairment' **AND **'cumulative structure' or 'scale construction' or 'guttman scaling' or mokken or rasch or uni-dimensional* or hierarch* or unidimensional* or IRT or 'item response theory' or 'patterns of functional decline' or scalogram or 'cumulative order' or 'one dimensional' or 'psychometric properties'.

**Figure 1:**
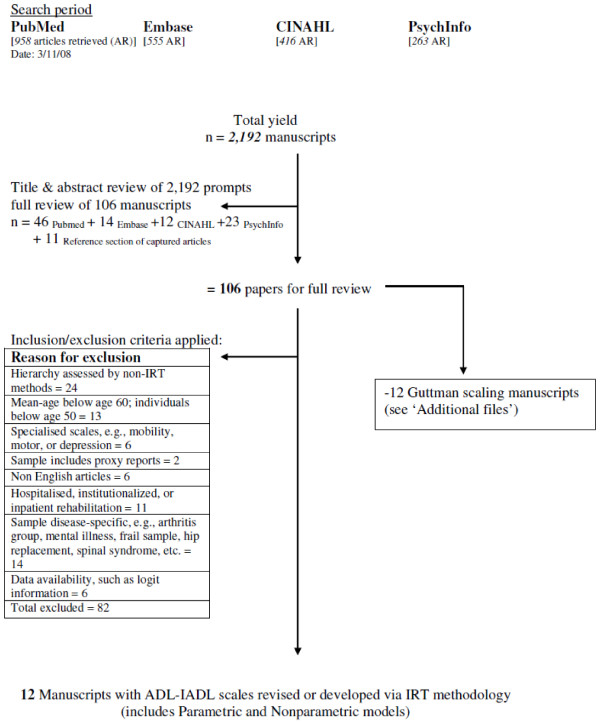
**Flow diagram for manuscript selection**. A systematic search of four databases (PubMed, Embase, CINAHL, and PsychInfo) resulted in the review of 2,192 manuscripts. Of these manuscripts, twelve met our inclusion/exclusion requirements and thus were targeted for further inspection.

Figure 1 depicts the flow chart for this review. After selecting 106 articles for full review, the reviewer examined the reference sections of these articles, which resulted in a total of 12 articles that required a full review. The initial search criteria included 'all languages'. Unpublished studies, dissertations, theses, book chapters or manuals, and studies published in non-peer-reviewed journals were not considered for the review.

### Inclusion and exclusion criteria

Generally, reports were included in this review if they described instruments with face validity for measuring disability, and thus closely reflect the fourth dimension of the Nagi [27] model (Difficulty doing activities of daily life, such as employment, household management, leisure activities, personal care, etc). The scales in this review will most likely resemble traditional Instrumental Activities of Daily Living [7], but will also, to a lesser degree, incorporate Basic or Personal Care Activities of Daily Living, as well as functional tasks (e.g., bending and kneeling, or walking outdoors). The latter more closely resembles the third dimension of the Nagi model. Scales were required to be generic measures, that is, should not be disease specific. The authors of this review chose to limit subject inclusion to those individuals 50 + years, with a sample mean age of 60 and above. Papers needed to scrutinize ADL-IADL performance with item response theory methods or Guttman scaling procedures. Reports that were primarily concerned with how broad domains of functioning, such as mobility, instrumental activities, and self-care activities form a hierarchy, while neglecting to assess item level functioning were not included. These types of studies, those targeting broad domains, presume a multidimensional structure to disability, and thus assess a hierarchy between domains. Manuscripts examining functional decline with a Medicare sample *were *included in this review, but were interpreted with caution, as these sample populations were generally more severely impaired than other community-dwelling samples. Studies using proxy reports were not included due to previous findings indicating a discrepancy between self-report and proxy ADL-IADL measures [28]. Despite the inclusion of manuscripts that utilised Guttman scaling procedures in our initial search criteria, in the end these manuscripts were excluded from the review. This was done for one of two reasons: 1) there is a large body of evidence asserting the inferiority of Guttman methods as compared to more advanced IRT procedures (see additional file 1); and 2) Many first generation functional status measures (i.e., Basic-ADLs) employed Guttman scaling procedures. Scales strictly examining Basic-ADLs are less relevant to this review because they are ineffective in assessing the early stages of disability in community-dwelling older adults.

In 2004 the Survey of Health and Retirement in Europe (spanning 11 European countries) indicated, for those aged 50 and over, that difficulty in at least one ADL task reached a high of 14% for Spain and a low of 7% in Switzerland [29]. In the same year, using data from approximately 20,000 subjects enrolled in Medicare, U.S. figures indicated that 12.6% of community-dwelling (aged 65 and over) older adults reported difficulty with at least one ADL task [30]. The problem with scales that restrict content to ADL items is that they cover a very limited range of health status. Even IADL measures designed to assess daily activities that were more complex than those assessed in the Katz ADL scale can present with large ceiling effects when applied to relatively healthy and or young older adults. Hardy et al. [31] indicates that, like ADL limitations, IADL limitations represent a fairly advanced stage of functional decline. Similarly, it was observed that decline in IADL usually begins after age 80 in community samples [32]. More recently, in a 4-year longitudinal sample purged of demented older adults, the magnitude of IADL decline was -.23 standard deviation per year [33]. It is important to note that mean baseline age for this sample was aged 78.

#### 1) Reliability

Scale reliability was measured in one of four ways: Item or Person Separation Index, Item or Person Separation Reliability, Test Information Function, and Rho Coefficient. The Test Information Function (TIF) represents the inverse of *standard error of estimation*. This standard error of estimation serves the same role as the standard error of measurement in classical test theory, except that the former statistic can vary for each examinee [24]. The TIF can be used to identify the most sensitive part of the instrument or scale [25]. *Item *reliability and separation statistics refer to the ability of the test to define a hierarchy of items along the measured variable, and the higher the number the more confidence we can place in the reliability of item placement across other samples or test administrations [34]. A similar principle applies to the *person *reliability and person separation index, i.e., replicability of person ordering and sufficient spread of person ability across the continuum. The reliability of item difficulty or person ability is interpreted on a 0 to 1 scale (similar to the way in which Cronbach's alpha is interpreted). These reliability estimates can be transformed to an item or person separation index, which reflects the number of standard errors of spread among the items or persons. Higher separation indicates a scale that covers a wider range of the construct being measured [34]. In assessing the separation index, the value should be at least 2 to obtain the desired reliability coefficient of .80. A *person *separation index of 2.0 indicates that the sample can be separated into at least three distinct groups [35], and an *item *separation index of 2.0 means that the items can be divided into three distinct levels of ability [36]. For the nonparametric Mokken scaling, Rho is used to define scale reliability, and is an internal consistency coefficient comparable to Cronbach's alpha [37]. Most theorists agree that a Rho over .80 is desirable, and a Rho over .70 is a minimum requirement [38].

#### 2) Validity

##### Construct validity

Of the four types of validity outlined by Cronbach and Meehl [39], this review will be most concerned with examining construct validities for each paper selected, as well as one aspect of content validity--namely, ceiling effects. An important aspect of construct validity is the trustworthiness of score meaning and interpretation [40]. It has been proposed that two major threats to score meaning and interpretation are *construct-irrelevant variance *and *construct under-representation *[41]. The former reflects unrelated sub-dimensions that are irrelevant to the construct being measured (e.g., disability), and the latter refers to the exclusion of important features of the construct (i.e., gaps in continuum coverage). Construct under-representation can be observed for parametric IRT models that provide interval level data. Because health status instruments are summed scores and typically include zero it has been common to treat them as continuous variables with ratio or interval characteristics. However, definition of a zero point is arbitrary and instrument dependent [42]. Furthermore, if the distance between items is not equally spaced, a segment change in an area of the scale with high item density will produce a greater numerical gain than a segment change in an area of the scale with low item density, despite the change being of equal magnitude. Typically, equally spaced interval units are derived by converting the raw score percentage into a success-to-failure ratio. Then the natural log of this odds ratio is computed.

Establishing a formal hierarchy of decline, or invariant item ordering (IIO), should enhance construct validity. In Likert scale models no strict item hierarchy is hypothesised or defined and priority is given to internal consistency [42]. With IIO, the order of the items in terms of difficulty should be the same for all respondents whatever their latent trait value [43]. Ligtvoet et al. [22] conveys that IIO is strong requirement in psychometrics, and that researchers wrongly assume that fitting any IRT model implies that the items have the same ordering by difficulty for all subjects. Furthermore, previous research has shown [44], rather surprisingly, that only restrictive polytomous IRT models provide IIO, i.e., rating scale models [45,46]. With regard to dichotomous-item tests, Sijtsma and Junker [43] demonstrated that the Rasch model [47] and the double monotonicity Mokken model [48] can also be used to establish IIO. The Mokken model for polytomous items also provides diagnostics for establishing IIO. When using the Mokken model, the criteria for IIO are met when the percentage of negative coefficients at the level of the individual subjects (H*^T^_a_*) is less than 10% and the coefficient for total set of subjects (H*^T^*) is at least .30 [49].

##### Content Validity

Content validity assesses whether the items measure what they claim to measure, and also if they measure the full range of the construct, which is discussed in terms of floor and ceiling effects [50]. These effects are the results of an item(s) clustering in the highest or lowest result group. The distribution of the results in the different review scales are presented and evaluated. The floor and ceiling effect is also considered important for the analysis of responsiveness. Floor and ceiling effects are presented in terms of responsiveness because they indicate limits to the range of detectable change, beyond which no further improvement or deterioration can be observed [50]. A maximum of 15% for any given sample has been proposed as the reasonable limit of ceiling or floor effects, with some investigators suggesting a ceiling threshold as low as 10% [51]. However, it has been observed that not all older adults become disabled, that is 20% of persons aged 95 and over have been shown to require no assistance with ADLs [52]. Thus, a figure below 15% might lead to questions concerning the validity of the construct we are intending the measure.

## Results

### Articles close to inclusion

Of the 106 articles selected for full review, six articles were excluded with some hesitation. Below is a list of articles that were very close to being included in the final list of 'review articles', but were ultimately excluded. All authors responded, but indicated that additional information was unavailable. 1) Avlund, Shult-Larsen, and Kreiner [53] was excluded due to data availability, specifically logit calculations and reliability coefficients. Avlund, Kreiner, and Shultz-Larsen [54] and Avlund, Kreiner, and Schultz-Larsen [55] were also excluded because logit information was unreported. McHorney [56] required reliability and item fit statistics for the community-dwelling sub-sample. In Finalyson, Mallinson, and Barbosa [57] the reliability coefficients, logit estimates, and fit statistics for community-dwelling subjects are not clearly separated from nursing home subjects or those receiving in-home services. Finally, for Cabrero-Garcia and Lopez-Pina [58] the analysis was solely conducted between gender groups. However, despite the insufficient information provided, several of these manuscripts will receive further attention in the discussion section of this manuscript.

Details of the twelve studies that met the full inclusion/exclusion criteria are listed below in Table 1. The table includes a number of factors thought to influence scalability, such as sample characteristics [59]. We chose to highlight, in bold type, the samples that were disproportionably female or male because gender has been shone to significantly affect item ordering [60].

**Table 1:** Studies using IRT to establish hierarchy of decline in ADL-IADL Scales

Study	ADL-IADL type	IRT model	# of items	Options	Sample studied	Reliability
Spector & Fleishman, 1998 (LH) [65]	National Long-Term Care Survey ADL & IADLs	Rasch-model	15 ADL- IADL (1 item removed)	2(disabled vs. not disabled) #	Representative sample of disabled in the community *, Age 65 +, M = 79; n = 2,977	PS Reliability: .88

Haley et al., 2002 [66]	Late-Life FDI(function component)	Rasch-Rating Scale	27 ADL & IADL (5 items misfit)	5 (assessing difficulty)	Community-dwelling, Age 60-98, M 75.9, SD 8.5; n = 150, **77% female**	IS Index: 10.1

Sheehan et al., 2002 [64]	NHEFS disability questionnaire	Rasch-Partial Credit	24 ADL-IADL (1 item misfit)	4 (assessing difficulty)	Noninstitutionalized general population of older Americans, Age 57-86, M = 62, n = 2,310	PS Index: 2.72 PS Reliability: .88

Jette et al, 2002 [67]	Late-Life FDI(disability component)	Rasch-Rating Scale	12 IADL (4 items misfit)	5 (assessing frequency)	Community-dwelling, Age 60-98, M 75.9, SD 8.5; n = 150	IS Index: 9.39

Fortinsky et al., 2003 (LH) [14]	Outcome and Assessment Information Set	Rasch-Partial Credit	15 ADL-IADL (zero items misfit)	3 to 6 (able to unable)	Community-dwelling, Medicare-eligible, with recent history of home care services, 1/3 of	Not reported

Dubuc et al., 2004 [62]	Physical Functioning Scale, PF -10	Rasch-Partial Credit	10 ADL-IADL (zero items misfit)	3 (limited by health)	Community-dwelling, n = 75, Age 60+, M 75.9, SD 8.5, **76% female**	TIF: 4.5

Schumacker, 2004 (LH) [16]	†	Rasch-Partial Credit	9 ADL- IADL (3 items removed)	2 (assessing fear)	Independent living facility (ILF), Age 65+, n = 91	IS Index: 3.01 PS reliability: .64

McHorney & Cohen, 2000 [69]	†	2-Parametric Graded Response Model	166 ADL-IADL items derived through test equating	6 (difficulty)	Veterans Association sample with **75% being male**, Age ≥ 65, n = 3358	Not reported

Kempen & Suurmeijer 1990 (LH) [38]	†	Mokken Scaling	18 ADL &IADL (zero item violations)	3 (difficulty)	Noninstitutionalized, Age 60 +, M = 74.5 n = 101, new users of prof. home help, **77% female**	Rho coefficient.: 0.96

Kempen et al., 1995 [59]	Groningen Activity Restriction Scale (short)	Mokken Scaling	12 ADL-IADL	2 (difficulty)	182 residents of seniors' apartments, M = 75, n = 182	Rho coeff.: 0.87

Kempen et al., 1996 [73]	Groningen Activity Restriction Scale (GARS)	Mokken Scaling	18 ADL-IADL (zero item violations)	4 (difficulty)	Commuity-based sample, Age ≥ 57, n = 4773	Rho coeff.: 0.93

Watson et al., 2010 [68]	Townsend Functional Ability Scale	Mokken Scaling	6 items (3 item violations)	3(difficulty)	Community-dwelling, All age 79, n = 548	Rho coeff.: 77

'number of items' reflects the ending point, i.e., hierarchy confirmed after IRT application; M = Mean Age of sample; SD = standard deviation; LH = least healthy samples; PS index = person separation index; IS index = item separation index; reliability = person separation reliability; * = disabled defined as needing assistance with at least 1 ADL-IADL task; † scale type unspecified or 'newly devised'; ILFs are located near nursing homes &/or retirement homes

#### 1) Reliability

A primary advantage of IRT is the extension of reliability. Traditionally, reliability (i.e., the degree to which a scale is free of measurement error) has been used to assess a scale's *average *reliability. IRT on the other hand, with the use of the information statistic researchers can determine how precise a scale is at various ranges of the latent trait [61]. Dubuc et al. [62] was the only manuscript to report a test information function, with a maximum score of approximately 4.5, which yields a standard error of .47. Despite the information curve being relatively flat and evenly distributed across the disability continuum, 4.5 is a rather modest value for this indicator of precision [63]. Hambleton [24] suggest that a TIF ≥ 10 is preferable. At any point on the latent variable, the standard error of a person estimate (on the complete set of items) is the inverse square-root of the TIF, so that a TIF of 10, person measure standard error = 0.32. Table 1 reports four different methods for assessing scale IRT-type reliability: Item or Person Separation Index, Item or Person Separation Reliability, Test Information Function, and Rho Coefficient. Several studies reported person reliability estimates, without reporting item reliability, i.e., Sheehan et al [64] and Spector and Fleishman [65] both reported a person reliability estimate of .88. These values indicate that the scale can differentiate persons on the measured variable (i.e., disability), and that one can place confidence in the reproducibility of placements. However, these values provide only half the picture, particularly if we are concerned with confirming a hierarchy of functional status items. Haley et al. [66] and Jette et al. [67] administered the Late-life FDI and recorded an item separation index of 10.1 and 9.39 respectively, which is well beyond the minimum requirement, and thus we can be confident that the scale provides an adequate number of statistically distinct difficulty strata with which to measure persons. Of the four manuscripts that employed Mokken scaling, all except Watson et al. [68], were far above the minimum requirement of 0.70. The Watson et al. functional status scale exceeded the minimum requirement for Rho, but fell short of the desired .80 mark.

One manuscript, Schumacker [16], that met the inclusion/exclusion criterion for this review was ultimately rejected (and not included in Table 1 below) because the reliability of this instrument was thought to be poor, so that score interpretation or inferences were impeded. The low person reliability value indicates that older adults are not responding in a consistent fashion across the set of 9 activity items for this scale. There appears to be an adequate person separation index, which means that there exists a large enough spread of ability across the sample so that the measures adequately reflect functional ability. However, the low person reliability suggests that the person ability estimates are not well targeted by the item pool. In most applications of IRT, reliability is estimated for both persons and for items. The Schumacker manuscript supports the utility of reporting both person and item statistics.

#### 2) Construct Validity

##### Construct under-representation

Seven scales from this review were able to establish interval level measurement using parametric IRT procedures. This enabled greater accuracy when considering change scores as well as identifying construct under-representation. All scales presented with relatively large gaps in coverage, with the exception of McHorney and Cohen [69]. Table 2 provides a summary of all the scales from this review that report interval level data. A relatively common method used to evaluate the distance between item calibrations is to perform a *t *test between successive pairs of items along the logit scale [34]. A gap in the item difficulty measure, which is defined as a significant *t *test for the difference between the measures of two successive items, is evidence of discontinuity in items [18]. However, when commenting on distances, one often needs to consider each authors definition of "difference" combined with their sample size and the structure of specific rating scales. And yet, some guidelines or standards have been proposed: a minimum spacing of .15 logits should ensure that items are distinct from each other [70], and a 'gap' beyond .30 logits might signal the need for additional items to avoid construct under-representation [71]. We limit our commentary of gaps to the percentage of interval space that exists between adjacent items.

**Table 2:** Scales establishing interval level data

Spector & Fleishmen, 1998	McHorney & Cohen, 2000	Sheehan et al., 2002	Jette et al., 2002
$\left(\begin{array}{c} \text{Shopping}\left(-.83\right)\\ \text{Doing laundry}\left(-.19\right) \end{array}\right\}\;26\%$	Scrub floor (1.75) Carry groceries 1 block (1.50)	$\left(\begin{array}{c} \text{Heavy house chores}\left(-2.49\right)\\ \text{Carry groceries}\left(-1.70\right) \end{array}\right\}\;15\%$	$\left(\begin{array}{c} \text{Active recreation}\left(62\right)\\ \text{Volunteer job}\left(53\right) \end{array}\right\}\;24\%$
Bathing (-.10)	Iron cloths (1.25)	Walk two blocks (-1.48)	Travel out of town (53)
Mobility outside (.02)	Stoop (1.00)	Light chores (-1.12)	Invite people to home (51)
Prepare meals (.29)	Cut toe-nails (.75)	Shop/run errands (-1.08)	Care for others (49)
Taking medicine (.38)	In/out of car (.50)	In/out bathtub (-1.02)	Visit friends & family (48)
Finances (.46)	Walk 1/2 block (.25)	Reach high, 5lb item (-.90)	Go out to public places (47)
Mobility inside (.53)	Wash dishes by hand (.00)	Wash hair (-.22)	Care of home, inside (42)
Light housework (.56)	Balance checkbook (- 0.25)	Arise from chair (-.19)	Take care of errands (41)
Dressing (.60)	Go to the bank (- 0.50)	Pick up cloths (-.13)	Keep contact w/others (36)
Transferring (.70)	Take vitamins (-0.75)	Up/down 2 steps + (-.12)	$\left(\begin{array}{c} \text{Take care of health}\left(33\right)\\ \text{Personal care needs}\left(25\right) \end{array}\right\}\;22\%$
Toileting (.94)	Wash face (-1.00)	Prepare own food (-.05)	
	Answer telephone (-1.25)	In/out of car (.10)	
$\left(\begin{array}{c} \text{Telephoning}\left(1.1\right)\\ \text{Incontinence}\left(1.60\right) \end{array}\right\}\;21\%$	Drink from a glass (-1.50)	Dress self + tie shoes (.24)	
Feeding (1.61)	**Fortinsky et al., 2003**	Wash & dry body (.33)	
**Haley et al., 2002**	Shopping (-3.35)	Open car doors (.45)	
	Laundry (-3.34)	Cut meat (.48)	
$\left(\begin{array}{c} \text{Run half mile}\left(75\right)\\ \text{Hike several miles}\left(65\right) \end{array}\right\}\;22\%$	Housekeeping (2.61)	Open milk carton (.49)	
Walk slippery surface (63)		Open jars (.56)	
Walk brisk mile (61)	$\left(\begin{array}{c} \text{Transport}\left(-1.87\right)\\ \text{Bathing}\left(-1.15\right) \end{array}\right\}\;10\%$	Write with pen or pencil (.59)	
Run to catch bus (60)	Prepare meals (-0.72)	Arise from bed (.75)	
Carry & climb stairs (59)	Dress lower (-0.02)	On/off toilet (.89)	
3 flights stairs inside (58)	Oral medication (.01)	Comb hair (1.17)	
1 flight outside (57)	Dress upper (0.56)		
Get up from floor (55)		$\left(\begin{array}{c} \text{Turn faucets on/off}\left(1.68\right)\\ \text{Lift full cup or glass}\left(2.75\right) \end{array}\right\}\;21\%$	
Walk one mile (53)	$\left(\begin{array}{c} \text{Grooming}\left(0.57\right)\\ \text{Ambulation}\left(1.64\right) \end{array}\right\}\;13\%$	**Dubuc et al., 2004**	
Walk several blocks (52)	Telephone use (1.78)		
Arise from low couch (51)	$\left(\begin{array}{c} \text{Toileting}\left(2.01\right)\\ \text{Transferring}\left(2.78\right)\\ \text{Feeding}\left(3.73\right) \end{array}\right\}\;13\%$	$\left(\begin{array}{c} \text{Vigorous activities}\left(66\right)\\ \text{Walk}1\text{mile}+\left(59\right) \end{array}\right\}\;21\%$	
On/off a bus (49)		Up several flights (58)	
Use step stool (48)		Bend, kneel, stoop (57)	
Open heavy door (47)		Walk several blocks (53)	
Up/down curb (46)		Lift or carry groceries(52)	
Bend over (45)		Moderate activities (50)	
1 flight of stairs inside (44)		Climb 1 flight (45)	
Reach overhead (43)			
Make bed (42)		$\left(\begin{array}{c} \text{Walk}1\text{block}\left(42\right)\\ \text{Bath or dress self}\left(32\right) \end{array}\right\}\;29\%$	
Get in/out of car (41)			
Pick up chair (40)			
Walking inside home (37)			
On/off coat (35)			
On/off trousers (34)			
Wash dishes (33)			
Hold full glass (30)			

Brackets indicate large gaps in coverage, as a percentage of the total disability continuum; Numbers in parentheses represent logit intervals, with some scales making a further conversion to a 0-100 range for increased ease in interpretation

The Spector and Fleishman scale [65] covers a logit range from -.83 to 1.61. There is a large gap in coverage between 'Shopping' and 'Doing laundry', which makes up 26% of the scale coverage. There is another gap (21% of the scale range) between 'Telephoning' and 'Incontinence help'. In Haley et al. [66] the coverage is relatively even, except for a large gap between the most difficult item, 'Run half mile', and the second most difficult item, 'Hike several miles'; the gap covers 22% of the scale. In Sheehan et al. [64] there exists one large gap between the two least difficult items, i.e. 'Lift a full cup or glass' and 'Turn faucets on and off'. The gap in coverage represents 21% of the scale range. There is another gap between the two most difficult items, which reflects 15% of to total scale coverage. In Fortinsky et al. [14] we find a 13% gap between 'Grooming' and 'Ambulation', a 13% gap between 'Transferring' and 'Feeding', as well as a 10% gap between 'Transport' and 'Bathing'. Dubuc et al. [62] records two large gaps at the top and bottom of the scale which occurs between 'Vigorous activities' and 'Walk one mile or more' (21% of the scale range), as well as between 'Walk one block' and 'Bath or dress self' (29%). Jette et al. [67] also records two large gaps in coverage, one between 'Active recreation' and 'Volunteer job' (range of 24%), as well as a gap between 'Personal care needs' and 'Take care of health' (22%). McHorney and Cohen [69] use the more complex 2-parameter scaling method, along with equating methods which allows for a large number of items (i.e., 166) to be placed on an interval scale. It is important to note that the Mokken scaling employs nonparametric procedures which do not produce a numerical estimate of item difficulty, but rather ranks items by the proportion of correct responses to an item.

##### Confirming a hierarchy

It should be noted that the number of scales that accurately report invariant item ordering is somewhat limited. This is because only two parametric models from this review are thought to imply invariant item ordering, the dichotomous Rasch model and the polytomous rating scale model [43,44]. The nonparametric Mokken model, when reporting the H^*T *^coefficient, is also capable of confirming IIO [72]. Table 3 below depicts scales that report invariant item ordering, thus formally confirming a hierarchy of functional decline. As expected, the Basic or Personal Care ADLs represented the least difficult items, or stated differently, difficulty with these items reflects the highest degree of subject severity. Interestingly, tasks that measure dexterity or fine motor skills (e.g., tie a knot or hold a glass) appear to reflect a greater level of severity than some personal care ADLs, such as bathing and dressing. Due to the limited number of scales from this review that are capable of establishing IIO, common items between scales were relatively few. However, if the 'Up and down stairs' item from Watson et al. [68] is most similar to the '3 flights of stairs inside' item from Haley et al. then we observe a common 3-item hierarchy for these two sales (i.e., stairs item followed by 'Get on a bus', followed by 'Reach overhead').

**Table 3:** Studies establishing invariant item ordering

Spector& Fleishmen, 1998	Haley et al., 2002	Jette et al., 2002	Watson et al., 2010 *
Shopping (-.826)	Run half mile (75)	Active recreation (62)	Cut toe-nails (.72)
Doing laundry (-.188)	Hike several miles (65)	Volunteer job (53)	Up/down stairs (.30)
Bathing (-.103)	Walk slippery surface (63)	Travel out of town (53)	Get on a bus (.22)
Mobility outside (-.022)	Walk brisk mile (61)	Invite people to home (51)	Reach overhead shelf (.16)
Prepare meals (.294)	Run to catch bus (60)	Care for others (49)	Wash all over (.09)
Taking medicines (.380)	Carry & climb stairs (59)	Visit friends & family (48)	Tie knot in string (.04)
Finances (.460)	3 flights stairs inside (58)	Go out public places (47)	
Mobility inside (.528)	1 flight outside (57)	Care of home, inside (42)	
Light housework (.559)	Get up from floor (55)	Take care of errands (41)	
Dressing (.597)	Walk one mile(53)	Keep contact w/others (36)	
Transferring (.699)	Walk several blocks (52)	Take care of health (33)	
Toileting (.944)	Arise from low couch (51)	Personal care needs (25)	
Telephoning (1.12)	On/off a bus (49)		
Incontinence help (1.60)	Use step stool (48)		
Feeding (1.61)	Open heavy door (47)		
	Up/down curb (46)		
	Bend over (45)		
	1 flight of stairs inside (44)		
	Reach overhead (43)		
	Make bed (42)		
	Get in/out of car (41)		
	Pick up chair (40)		
	Walking inside house (37)		
	On/off coat (35)		
	On/off trousers (34)		
	Wash dishes (33)		
	Hold full glass (30)		

All scales present most difficult items first; * = scales assessed through nonparametric procedures; Numbers in parenthesis = logit values

#### 3) Content Validity

Four of the twelve scales were exceptional in reducing ceiling effects: Kempen and Suurmeijer [38] reported 5% of subjects at the ceiling level; Fortinsky et al. [14] also reported a ceiling effect of 5%; Haley et al. [66] and Jette et al. [67] observed a ~1% and 0% ceiling effect, respectively. However, it would appear that the success of Kempen and Suurmeijer and Fortinsky et al. has more to do with sample characteristics than item or task difficulty. Both scales were categorised in Table 1 as having the 'least healthy' samples of older adults. This line of reasoning is confirmed by the fact that the bathing personal care ADL appears in the top 3^rd ^of most difficult items for the Fortinsky et al. scale. Similarly, in the Kempen and Suurmeijer scale 'climbing a flight of stairs inside' appears in the top 3^rd ^of most difficult items, but this is a relatively easy mobility items when compared to the mobility hierarchy presented in Haley et al. [66].

With the exception of Schumacker [16] which found that 70% of their older adults reported an inability to perform 7 out of 9 activities due to fear, most of the floor effects were negligible. Thus, our results are primarily concerned with the identification of ceiling effects. Kempen et al. [59] found that 85% of the sample could manage the most difficulty item, 'Going up & down stairs'. Spector and Fleishman [65] began their study by restricting their sample to those subjects that were functionally disabled in at least one task (4463 to 2977). Thus the ceiling could be considered to include 32% of subjects, which was very similar to that reported in Watson et al. [68] (33%). Kempen et al. [73] reported ceiling effects for 44.8% of the sample (n = 2144) and 8.4% of the sample (n = 403) scored ≥ 36 on the GARS (theoretical range of 18-72). Sheehan et al. [64] also reported a very large ceiling effect, n = 2079 (46.9%). Dubuc et al. [62] indicated a ceiling effect of 16%. McHorney and Cohen [69] reported that ~ 15% of their subjects had no difficulty with the six largest location parameter estimates, i.e., the 6 most difficult items. Fortinsky et al. [14] and Kempen & Suurmeijer [38] reported similar ceiling effects. In Fortinsky et al., 5% of subjects reported no disability, and for Kempen & Suurmeijer 5% of subjects reported no problems with the most difficult item. Jette et al. [67] and Haley et al. [66] recoded the lowest levels of ceiling and floor effect which outperformed the proposed standards [51], with 0% and ~ 1% respectively.

## Discussion

This review was concerned with the evolution and enhancement of ADL-IADL scales that specifically target high functioning community-dwelling older adults. It has been proposed that the relative standing of self-report ADL-IADLs could be enhanced by improving construct validities that are at least equivalent to those of physical performance measures. To address these challenges, this review chose to investigate constructs related to scale hierarchy, ceiling effects, and establishing interval level measurement that enables the identification of construct under-representation.

Seven scales from this review were able to establish interval level measurement using parametric IRT procedures, thus enabling greater accuracy when considering change scores as well as identifying construct under-representation. With regard to construct under-representation all scales in this review presented with relatively large gaps in coverage, with the exception of McHorney and Cohen [69]. When IRT methods are used to transform the ordinal nature of ADL scales to interval level data, diagnostic precision [15] and sensitivity to clinical change are enhanced [74]. Comparing disability measurements between patients, or within patients between different moments in time is complicated. Change scores for Likert summative scores need to be interpreted with caution. It has been noted that assessing change in terms of estimated trait level rather than raw scores can yield more accurate estimates of change [75]. If non-equal intervals exist between adjacent items, change scores for subjects with different levels of ability may misrepresent the amount of change, or fail to detect change in the latent trait [51]. Furthermore, Fraley et al. [76] demonstrated that analyses of change at the raw-score level and analysis of change using the latent-trait metric may lead to opposite conclusions. In one example, they displayed results showing that highly anxious individuals are relatively less stable over time when considered at the raw-score level, but more stable over time when considered at the latent-trait level. Thus, failing to understand the scaling properties of an instrument can lead to grossly inaccurate conclusions [77].

Four scales met IRT standards for ascertaining item hierarchy at the individual level, as opposed to merely establishing item hierarchy at the population level. Despite the comprehensive coverage of McHorney and Cohen [69], this manuscript made use of the 2PL IRT model which does not provide the added advantage of invariant item ordering; Ligtvoet et al. [22] point out that Sijtsma and Hemker [44] proved that the graded response model used in McHorney and Cohen does not imply invariant item ordering. Invariant item ordering is clinically useful because improved understanding of the sequence of functional change or decline and its natural trajectory in aging would open up opportunities for thinking about early intervention and/or ways to change this trajectory [20,78]. Ligtvoet et al. [22] reports that IIO is a strong requirement in measurement practice, and that researchers sometimes assume that fitting an IRT model implies that items have the same ordering by difficulty or popularity for all individuals, but this assumption requires modification. In following this rather strict criterion for IIO, our final pool of scales was relatively limited. This resulted in very few items that were common to other scales, thus allowing for only modest patterns of functional decline to emerge.

It has been noted, within the last 25 years, that interest in measuring functional status among the nondisabled elderly has expanded dramatically because of the aging of the population and its implications for health care policy. As a result, measures of ADLs and IADLs have increasingly been applied to community-dwelling individuals, resulting in substantial ceiling effects [79]. Four of the twelve scales were exceptional in reducing ceiling effects: Kempen and Suurmeijer [38] reported 5% of subjects at the ceiling level; Fortinsky et al. [14] also reported a ceiling effect of 5%; Haley et al. [66] and Jette et al. [67] observed a ~1% and 0% ceiling effect, respectively. However, it should be considered whether the success of the scales used in Kempen and Suurmeijer as well as Fortinsky et al. are being driven more by sample characteristics than scale sensitivity. Both scales were categorised in Table 1 as having the 'least healthy' samples of older adults. The Kempen and Suurmeijer sample were all new users of professional home help, in addition to subjects being 77% female. Again, gender should be considered, as previous studies have reported gender differences in functional disability, with elderly women reported to have higher functional disability than elderly men [80]. The Fortinsky et al. sample were described as Medicare-eligible with a recent history of home care services, and one third of the sample was age 85 or above. Despite Haley et al. and Jette et al. also having large proportion of female subjects, their subjects appear much healthier than the two other samples mentioned above. Thus, we are more confident that the low percentage of ceiling effects has more to do with scale characteristics.

The success related to improved content validity can be attributed to the development of more difficult items. The items used in Haley et al. [66] are very different than traditional IADL items (e.g., assessing the ability to 'Run a half mile'). In an effort to approach the novel status of a 0% ceiling effect, Haley et al. increased item difficult. However, it has become apparent that 'newly developed' items designed to limit ceiling effects in high functioning populations lie outside the realm of daily experience, and thus may prove less reliable. For instance, questions about walking difficulty over a distance of one-quarter mile or more may be answered inaccurately simply because the respondent has not attempted to walk such a distance in quite some time [81]. Furthermore, it has been noted that the 'Vigorous activities' item (from a sample of chronically ill or psychiatric subjects) may have misfit due to lack of actual engagement in these activities within a typical day [82].

Lawton's instrumental activities of daily living [7] were thought to reflect a greater degree of complexity than the previously developed ADLs, and thus would be more applicable to a broader population of older adults. However, it seems that these traditional IADLs are most responsive to community dwelling older adults that show early sings of cognitive pathology, such as mild cognitive impairment. It has been shown that a majority of the traditional IADLs are more closely approximated with physical fitness than cognitive complexity [83]. In an effort to reduce ceiling effects and to track change in community-dwelling older adults, scale developers have chosen to assess tasks that are more and more physically demanding, e.g., 'Run a half mile' or 'Vigorous activities'. However, the Late Life FDI scale presented in Jette et al. [67] utilises difficult items (as evidenced by a ceiling effect of 0%), while maintaining a degree of complexity, e.g., the 'Travel out of town' item or 'Invite people into home'. And yet this scale does have two relatively large 'gaps' in coverage that might make tracking change over time problematic. Also these sorts of items may prove cumbersome for tracking progress in 'prehabilitation' (e.g., cognitive training) over relatively short intervention periods. It might be fruitful to explore the embedded components of a complex task such as 'Travel out of town', much the same way geriatricians have scrutinised the sub tasks involved in bathing [84,85].

Another avenue for increasing scale sensitivity in community-dwelling older adults is to alter the wording and thus the context in which activities are performed. Fries et al. [86] provides a review (with a mixed patient population) on the effects of altered context. In this review, Avlund et al. [54], like Jette et al. [67], explored atypical disability wording in an effort to reduce ceiling effects in community-dwelling populations (Avlund et al. is cited in the 'close to inclusion' section of this manuscript). Avlund et al. [53] compared 'tiredness' and 'reduced speed' classifications, and found that the reduced speed scale was more effective in reducing ceiling effects. However, Avlund et al. [55] advocated the rejection of the reduced speed scale (in favour of the 'tiredness') due to severe heterogeneity across age groups, as well as model fit difficulties. Avlund et al. [55] also compared dependency (i.e., 'do you need help?') vs. tiredness and found that the tiredness scale was more suitable for measuring change among well older adults. At the same time, Fried et al. [87] were altering scale classification by asking whether health or physical problems result in ADL-IADL tasks being completed with less frequency, or do such problems cause individuals to modify how they perform a particular functional task. Lastly, from this review, Schumacker [16] used the uncommon categorization of 'Do you have fear?' performing various ADL-IADL activities. The result was massive floor effects, and the manuscript was ultimately excluded from this review because of poor reliability. It's worth mentioning that the categorization or wording of a particular ADL-IADL item (i.e., the differences that often occur between large surveys or cohorts) can prevent data from being pooled to create much bigger samples with increased statistical power. This topic lies beyond the scope of this review, but it should be noted that IRT equating procedures can be used to bring different groups together for comparisons on a common scale. The potential for such methods can be seen in Jagger et al. [88] in which there was a desire to make disability comparisons between five national surveys.

A primary advantage of IRT is the extension of reliability. Traditionally, reliability (i.e., the degree to which a scale is free of measurement error) has been used to assess a scale's *average *reliability. IRT however, is able to evaluate measurement error, or precision, at various stages along the scale continuum (e.g., disability construct). This is valuable because precision along the continuum is not uniform, and thus is expected to vary. This information is summarized with the information function, which allows for the estimation of the standard error of measurement for each subject's ability level. Despite the obvious utility, only one manuscript from this review chose to estimate the test information statistic--namely, Dubuc et al. [62].

Our review contains only one 2PL manuscript, which could be viewed as a study limitation. Some authors have suggested that 1PL models, as compared to 2PL models, are unsuitable as a final model for describing data resulting from functional status items [21]. Similarly, the fit of an IRT model can be examined with a likelihood ratio test, which assumes the more parameters that are used to describe item and subject behaviour, the better the model will fit the data [56]. However, the 1PL model is more robust [21] and has the advantage of assuring that items can be ordered unambiguously, in the sense that their item characteristic curves do not cross [65] The 1PL procedure is the only well-known parametric IRT (as well as the rating scale model for polytomous items) model that has nonintersecting IRFs [72]. Additionally, the item fit statistics available for the 2PL model are barely reliable for scales containing few items and very sensitive with large samples [58]. A further limitation relates to the unavailability of data. This resulted in some logit data being extracted from figures rather than tables. This will merely have a small impact on the accuracy of reporting. Finally, several studies in this review use less than 100 subjects in their IRT analyses, which may be small even by Rasch standards. It has been proposed that a sample size of 100 will provide 95% confidence of item calibration. However, it has also been suggested that the adequacy of test targeting influences sample-size, and thus, a well-targeted test may produce adequate location precision with less than 100 subjects [51].

## Conclusion

This manuscript sought to meet the challenge associated with identifying an early functional state of disability by improving instrument calibration. Traditional disability instruments, when applied to relatively high functioning older adults, poorly discriminate, as well as underestimate disability in the early stages of development. Poor discrimination refers to tasks or activities (i.e., scale items) that prove unresponsive to changes in a particular person's ability level. Item response theory (IRT) methodology can be used to improve the structure of ADL-IADL scales so that they are more sensitive in detecting preclinical stages of functional decline within community-dwelling older adults, a stage that has been shown to be more responsive to clinical interventions aimed at prevention of overt disability or frailty. This review sought to demonstrate that the calibration of ADL-IADL can serve to identify individuals at high risk for future disability, potentially years before clinical onset. IRT models can increase the interpretive power of ADL-IADL scales in multiple ways: by confirming an underlying uni-dimensional continuum of disability, establishing interval level measurement or item hierarchies, and increasing scale precision.

## Competing interests

The authors declare that they have no competing interests.

## Authors' contributions

All authors have read and approved the final manuscript. RF, IJD, EJA and JMS have taken part in designing and planning of the study, as well as editing pre-submission drafts of this manuscript. RF conducted data collection.

## Pre-publication history

The pre-publication history for this paper can be accessed here:

http://www.biomedcentral.com/1471-2318/11/42/prepub

## Supplementary Material

Additional file 1 **Guttman scaling has become less relevant**. This section is meant to explain why the authors chose not to include manuscripts that employed Guttman scaling. Click here for file
