# Supplementary material for: Calibrating ADL-IADL scales to improve measurement accuracy and to extend the disability construct into the preclinical range: a systematic review
Source: BMC Geriatr. 2011 Aug 16;11:42. doi: 10.1186/1471-2318-11-42 (PMC3201016; doi:10.1186/1471-2318-11-42)
Supplement: Additional file 1 — Guttman scaling has become less relevant. This section is meant to explain why the authors chose not to include manuscripts that employed Guttman scaling. [file 1471-2318-11-42-S1.DOC]

**Additional files**

Due to the Guttman model’s [1] extensive use in geriatric research for a period of approximately two decades, the authors of this review thought it pertinent to include functional status measures that were scaled in this way. However, during this systematic review, it readily became apparent that the Guttman methods were somewhat unreliable in formally confirming a hierarchy of functional status. As early as the 1980s researchers began highlighting serious shortcomings in the Guttman scaling procedure applied to ADLs. Some of the criticisms included: **1)** Guttman is a deterministic model based on the expectation that persons will pass all items that are easier than their ability level and will fail all items that are more difficult. But in reality, answer patterns are subject to more than the underlying construct or trait; answer patterns will also be influenced by other factors such as interpretation of the question and mood of the respondent. Therefore, the relationship to item responses and the construct is better framed as probabilistic rather than deterministic [2]. To ensure that a scale conforms to this expectation of clear-cut pass/fail point for each person, the differences between item difficulties must be large [3]. Thus, the sensitivity of such scales to small changes in functioning within individuals over time or to small differences between individuals is dramatically reduced [4]. Also, the deterministic quality of Guttman reduces the potential to accurately define measurement error. Stated differently, the Item Characteristic Curves of this model will be a perfect step function, which is problematic because researchers will have difficulty determining whether deviations from the proposed item hierarchy represent error or that the construct is not sufficiently unidimensional [5]. **2)** Rigid hierarchies expected by Guttman scales have rarely been borne out in either social or behavioural research [6;7]; ‘…the use of Guttman scales has been found to be severly compromised by the problem of large numbers of response patterns that do not conform to the Guttman requirements’ [8, p.87]. **3)** Sheehan et al. [9] reported: Lazaridis and colleagues [10] studied the scalability of selected ADL items using criteria associated with Guttman scales and found that the Katz ADL fulfilled Guttman’s scaling criteria. However, Lazaridis et al. also found that the Katz hierarchy was one of 360 possible hierarchies, based on permutations of six ADL items. Lazaridis tested all 360 of these hierarchies using the same Guttman scaling criteria and found four additional scoring schemes that performed equally as well as Katz, and found a total of 103 scoring hierarchies that satisfied the minimum standards of scalability according to Guttman. ‘The fact that there is not a single hierarchical scale, but as many as 103 different hierarchies underlying Katz’ six original ADL items, exposes the disadvantage of a rigid and deterministic hierarchy’ [9, p.843].

As a consequence of the criticisms noted above, in addition to the development of more sophisticated probabilistic methods (i.e., Item Response Theory), Guttman scaling procedures in health science research have for the most part been abandoned [11]. However, exceptions do exist, with some contemporary research implementing Guttman scaling [12]. Due to the points outlined above, the inclusion of ‘Guttman hierarchies’ in this systematic review was limited to the initial search strategy. That is, an examination of the references sections of accepted manuscripts excluded the identification and extraction of Guttman-type papers, e.,g Lawton & Brody [13].

1. Guttman L. **The basis for scalogram analysis**. In: S. A. Stouffer & et al. (Eds.). Studies in social psychology in World War II. New York: Wiley; 1950:60-90. Measurement and prediction.
2. Kempen G, Myers A, Powell L. **Hierarchical structures in ADL and IADL: analytical assumptions and applications for clinicians and researchers.** *J Clin Epidemiol* 1995, **48**:1299-1305.
3. Fisher WP, Fisher AG. **Applications of Rasch analysis to studies in occupational therapy.** *Phys Med Rehabil Clin N Am*: *New Developments in Functional Assessment* 1993, **4**: 551-569.
4. Finch M, Kane RL, Philip I. **Developing a new metric for ADLs.** *JAGS* 1994, **43**: 877-884.
5. Gillespie M, Tenvergert EM, Kingma J. **Using Mokken scale analysis to develop unidimensional scales.** *Qual Quant* 1987, **21**:393–408.
6. Siu AL, Reuben DB, Hays RD. **Hierarchical measures of physical function in ambulatory geriatrics.** *JAGS* 1990, **38**:1113-1119.
7. Wilson M. **A comparison of deterministic and probabilistic approaches to measuring learning structures.** *AJE* 1989, **33**:127-144.
8. Wilson M: *Constructing Measures: An Item Response Modelling Approach*. Mahwah, NJ: Erlbaum; 2005.
9. Sheehan TJ, DeChello LM, Garcia R, Fifield J, Rothfield N, Reisine S. **Measuring disability: application of the rasch model to activities of daily living (ADL/IADL).** *J Outcome Meas* 2002, **5**:839-863.
10. Lazardis EN, Rudberg MA, Furner SE, Cassel CK. **Do activities of daily living have a hierarchical structure? An analysis using the longitudinal study of aging.** *J Gerontol* 1994, **49**:M47-M51.
11. Vittengl JR, White CN, McGovern RJ, Morton BJ. **Comparative validity of seven scoring systems for the instrumental activities of daily living scale in rural elders.** *Aging Ment Health* 2006, **10**: 40-47.
12. LaPlante MP. **The classic measure of disability in activities of daily living is biased by age but an expanded IADL/ADL measure is not.** *J Gerontol B Psychol Sci Soc Sci*. (In Press).
13. Lawton MP, Brody EM. **Assessment of older people: self-maintaining and instrumentalactivities of daily living.** *Gerontologist* 1969, **9**:179-186.
